# *Baccharis trimera* (Carqueja) Improves Metabolic and Redox Status in an Experimental Model of Type 1 Diabetes

**DOI:** 10.1155/2018/6532637

**Published:** 2018-12-04

**Authors:** Natália Nogueira do Nascimento Kaut, Ana Carolina Silveira Rabelo, Glaucy Rodrigues Araujo, Jason Guy Taylor, Marcelo Eustáquio Silva, Maria Lúcia Pedrosa, Miriam Martins Chaves, Joamyr Victor Rossoni Junior, Daniela Caldeira Costa

**Affiliations:** ^1^Programa de Pós-graduação em Saúde e Nutrição, Escola de Nutrição, Universidade Federal de Ouro Preto (UFOP), Ouro Preto, MG, 35400-000, Brazil; ^2^Programa de Pós-graduação em Ciências Biológicas, NUPEB, Universidade Federal de Ouro Preto (UFOP), Ouro Preto, MG, 35400-000, Brazil; ^3^Laboratório de Célula-tronco, Departamento de Anatomia de Animais Domésticos e Silvestres, Faculdade de Medicina Veterinária e Zootecnia, Departamento de Cirurgia (VCI), Universidade de São Paulo (USP), São Paulo, 05508-270, Brazil; ^4^Departamento de Química, Universidade Federal de Ouro Preto (UFOP), Ouro Preto, MG, 35400-000, Brazil; ^5^Departamento de Alimentos, Escola de Nutrição, Universidade Federal de Ouro Preto (UFOP), Ouro Preto, MG, 35400-000, Brazil; ^6^Departamento de Ciências Biológicas, Instituto de Ciências Exatas e Biológicas, Universidade Federal de Ouro Preto (UFOP), Ouro Preto, MG, 35400-000, Brazil; ^7^Departamento de Bioquimica e Imunologia, Instituto de Ciências Biológicas, Universidade Federal de Minas Gerais (UFMG), Belo Horizonte, MG, Brazil

## Abstract

Diabetes mellitus is a metabolic disorder that causes severe complications due to the increased oxidative stress induced by disease. Many plants are popularly used in the treatment of diabetes, e.g.,* Baccharis trimera* (carqueja). The aim of this study was to explore the potential application of the* B. trimera* hydroethanolic extract in preventing redox stress induced by diabetes and its hypoglycemic properties. Experiments were conducted with 48 female rats, divided into 6 groups, named C (control), C600 (control + extract 600 mg/kg), C1200 (control + extract 1200 mg/kg), D (diabetic), D600 (diabetic + 600 mg/kg), and D1200 (diabetic + 1200 mg/kg). Type 1 diabetes was induced with alloxan, and the animals presented hyperglycemia and reduction in insulin and body weight. After seven days of experimentation, the nontreated diabetic group showed changes in biochemical parameters (urea, triacylglycerol, alanine aminotransferase, and aspartate aminotransferase) and increased carbonyl protein levels. Regarding the antioxidant enzymes, an increase in superoxide dismutase activity was observed but in comparison a decrease in catalase and glutathione peroxidase activity was noted which suggests that diabetic rats suffered redox stress. In addition, the mRNA of superoxide dismutase, catalase, and glutathione peroxidase enzymes were altered. Treatment of diabetic rats with* B. trimera *extract resulted in an improved glycemic profile and liver function, decreased oxidative damage, and altered the expression of mRNA of the antioxidants enzymes. These results together suggest that* B. trimera* hydroethanolic extract has a protective effect against diabetes.

## 1. Introduction

Diabetes mellitus (DM) is a metabolic disorder caused by dysfunction in the secretion and response to insulin, leading to increased blood glucose levels (hyperglycemia) [1–3]. Globally, approximately 415 million people suffer from diabetes, and one person dies every six seconds with this disease [4]. It is estimated that the number would be 642 million people by 2040 [5]. Diabetes is accompanied by severe oxidative stress, which is caused by abnormal metabolism induced by hyperglycemia that can result in the overproduction of reactive oxygen species (ROS) [3, 4]. ROS level elevation in diabetes may be promoting glycation of proteins, glucose oxidation and increased lipid peroxidation leads to damage of enzymes, cellular machinery, and also increased insulin resistance due to oxidative stress [3]. These conditions lead to development of diabetic complications such as nephropathy, retinopathy, neuropathy, and micro- and/or macrovascular injuries [4, 6]. Therefore, antioxidants have been shown to play a beneficial role in the prevention of complications associated with diabetes [3, 7]. A variety of plant extracts have been used for centuries in folk medicine to treat various diseases. Medicinal plants are particularly interesting because not only can they be used as complementary remedies to prevent metabolic diseases, but they also serve as an interesting source of potential drug candidate compounds [8]. Several plant species have demonstrated anti-diabetic properties, and a large number of compounds from plant extracts have been reported to have beneficial effects in the treatment of diabetes [9]. A listed 1200 species of plants that have been used to treat diabetes worldwide; among these are the species of the Asteraceae family [10]. The* Baccharis *genus of the Asteraceae family is distributed mainly in Brazil, Argentina, Colombia, Chile, and Mexico [11].* Baccharis trimera* (Less.) D.C., popularly known as “carqueja,” is one of the most commonly used medicinal plant and already had several proven biological effects such as hypoglycemic [12], hepatoprotective [13, 14] and antioxidant [15–17]. Other species of the genus* Baccharis *may present marked toxicity [18, 19], raising concerns about the use of plants from this genus. Studies on the chemical composition of* B. trimera* demonstrated that this plant has many bioactive compounds, such as flavonoids [17], diterpenes, and triterpenes [20]. Flavonoids are well-known for their multiple biological functions, including antidiabetic [21, 22]. Therefore, the aim of this study was designed to explore the capacity of the* B. trimera* hydroethanolic extract to protect against oxidative stress induced by diabetes and to determine its hypoglycemic properties.

## 2. Materials and Methods

### 2.1. Collection of Plant Material and Preparation of Extract

The aerial parts of* B. trimera *were collected in the city of Ouro Preto, Minas Gerais, Brazil. The specimen, voucher number OUPR 22.127, was identified and deposited in the Herbarium José Badini–UFOP. After identification, the aerial parts of the plant were dried in a ventilated oven, sprayed in a mechanical mill and stored in plastic bottles. To obtain the hydroethanolic extract, approximately 100 g of powdered, dried aerial parts of the plant were submitted to extraction with a 100 mL ethanol (70%) water solution (1:1 v/v) at room temperature for 24 h. Vacuum filtration and elimination of the solvent in a rotary evaporator were then performed. The crude extract obtained (from 100 g) was then diluted with distilled water. Concentrations of 600 and 1200 mg/kg body weight were used* in vivo*. The methodology for the extract preparation was based on the work of Grance et al. 2008 [23], with some modifications. A phytochemical characterization study of the* B. trimera* hydroethanolic extract was carried out by our group [15, 16]. Flavanoides quercetin and rutin were encountered in the hydroethanolic extract of* B. trimera *utilized in this study.

### 2.2. DPPH Radical-Scavenging Activity

The DPPH radical-scavenging activity of the* B. trimera *extract was determined using a modified method [22]. The antioxidant activity was determined from the reduction in the absorbance of the DPPH radical at 515 nm.

### 2.3. Cytotoxic Analysis (*Artemia salina*)

The cytotoxicity was conducted using the brine shrimp lethality test [24], a widely used bioassay. The crude fraction of the plant extract was used for the test (LC_50_). Probity analysis was used to determine the lethal concentration (LC_50_) of* B. trimera* hydroethanolic extract on nauplii.

### 2.4. Animals

The Laboratory of Experimental Nutrition at the Federal University of Ouro Preto (UFOP) provided the female albino Fischer rats used in the experiment. The animals were approximately 12 weeks old and weighed approximately 180 g. All animals were kept in collective cages (five per cage), placed in an environment with controlled temperature, light, and humidity, and given both commercial rat chow and water* ad libitum*. This work was conducted in accordance with the international standards of animal protection and with the ethical principles of the Brazilian College of Animal Experimentation, and the protocols were approved by the Ethics Committee on Animal Use (CEUA) of UFOP (OF 166/2011 protocol 2011/82).

### 2.5. The Experimental Protocol

Forty-eight female rats were distributed into six groups according to the treatment they received:

- Control group (C) received water twice daily (08:00 and 18:00 h).
- Control treated group 600 mg/Kg (C600) received 600 mg/kg of the* B. trimera* hydroethanolic extract twice daily (08:00 and 18:00 h).
- Control treated group 1200 mg/Kg (C1200) received 1200 mg/kg of the* B. trimera* hydroethanolic extract twice daily (08:00 and 18:00 h).
- Untreated diabetic group (D) received water twice daily (08:00 and 18:00 h).
- Diabetic treated group 600 mg/Kg (D600) received 600 mg/kg of the* B. trimera* hydroethanolic extract twice daily (08:00 and 18:00 h).
- Diabetic treated group 1200 mg/Kg (D1200) received 1200 mg/kg of the* B. trimera* hydroethanolic extract twice daily (08:00 and 18:00 h).

All treatment were administrated by gavage, with the first dose administered 72 h after the induction of diabetes. After 7 days of treatment food was removed and they were euthanized by deep anesthesia induced by isoflurane. The dose* B. trimera* extract was based on previous work [15].

### 2.6. Induction of Experimental Diabetes

Diabetes was induced by an intraperitoneal injection of 135 mg/kg alloxan (ALX) dissolved in 0.2 mL aqueous solution of NaCl (0.01 M, pH 4.5). The nondiabetic animals received an intraperitoneal injection of NaCl. Three days after the administration of ALX, blood samples were collected by the tail vein, and glucose was measured using Accu-Chek active (Roche Laboratories, San Francisco, USA) to confirm the development of diabetes. After overnight fasting, animals with glucose levels above 300 mg/dL (16.65 mmol/L) were considered diabetic. At the end of the experiment, biochemical analyses of plasma glucose concentrations and insulin levels were determined using the Kits Labtest (Lagoa Santa, MG, Brazil) and the Ultra-Sensitive Rat Insulin Elisa Kit (Crystal Chem, Downers Grove, IL, USA).

### 2.7. Biochemical Parameters

For biochemical analysis, urea, creatinine, triacylglycerol (TAG), cholesterol, alanine aminotransferase (ALT), and aspartate aminotransferase (AST) were determined in serum by the Kits Labtest (Lagoa Santa, MG, Brazil).

### 2.8. Preparation of Liver Tissue

Liver tissue was collected immediately after euthanasia of the animals. The tissue was homogenized to determine the concentrations of oxidative stress markers, enzymes activities, and real-time quantitative RT-PCR assay.

#### 2.8.1. Determination of Oxidative Stress Biomarkers

*(1) Thiobarbituric Acid Reactive Substances (TBARS)*. TBARS concentration was determined from thiobarbituric acid (TBA) binding to oxidized lipids. This measurement was performed according to [25]. Values were expressed in nmoles per mg of protein.

*(2) Carbonyl Protein*. Protein oxidation by reactive oxygen species (ROS) leads to the formation of carbonyl derivatives, which can be measured by sensitive methods. Measurements of carbonyl protein were performed according previously [26]. The results were expressed as nmol of carbonyl groups per mg of protein.

#### 2.8.2. Determination of Antioxidant Defenses

*(1) Catalase (CAT)*. Catalase activity was determined based on its ability to convert hydrogen peroxide (H_2_O_2_) into water and molecular oxygen. The assays were performed as described [27]. One unit (U) of catalase has a hydrolytic capacity equivalent to 1 *μ*mol of H_2_O_2_ per minute.

*(2) Superoxide Dismutase Activity (SOD)*. The activity of total superoxide dismutase (SOD) was measured using a kit (Cayman Chemical Company, MI, USA). The reaction was initiated by adding xanthine oxidase, and the absorbance was measured at 450 nm using a plate reader (Biotek ELx808). 

*(3) Total Glutathione Concentration*. Glutathione is present in cells mainly in its reduced form (GSH), which represents approximately 90% of the total glutathione in the cell. The remaining amount is in the form of oxidized glutathione (GSSG). To determine the levels of total glutathione (GSH+GSSG) in our biological samples, we used a Sigma kit (CS0260). Total glutathione was expressed in nmoles per ml of sample. 

*(4) GSH and GSSG Content*. The procedure to measure the levels of GSSG was the same as that used to measure total glutathione, but the GSH content of the sample was first depleted by a derivatization process using 2-vinylpyridine (Sigma-Aldrich, St. Louis, MO, USA). It was possible to calculate the GSH content by subtracting the GSSG from total glutathione [28]. 

*(5) Glutathione Peroxidase Activity (GPx)*. The Glutathione Peroxidase Cellular Activity Assay kit (Sigma-Aldrich,St. Louis, MO, USA) was used to measure glutathione peroxidase activity in tissue extracts. The enzyme activity was expressed as units/ml. 

*(6) Glutathione Reductase Activity (GR)*. Glutathione reductase (GR) activity was measured using a kit (Sigma-Aldrich, St. Louis, MO, USA). The enzyme activity was expressed as units/ml.

#### 2.8.3. Real-Time Quantitative RT-PCR Assay

Total RNA was isolated from 50 mg of liver tissue of rats using the RNAgents Total RNA Isolation System (Promega Corporation, Madison, WI) according to the manufacturer's instructions. The concentration and purity of RNA were estimated spectrophotometrically from the A260/A280 ratio (NanoVue, GE Healthcare, UK). cDNA was synthesized from 2 *μ*g of total RNA with random primers using the High-Capacity cDNA Reverse Transcription Kit (Applied Biosystems, Foster City, CA, USA) following the manufacturer's recommendations.

Quantitative real-time PCR (qPCR) was performed using the* Power* SYBR® Green PCR Master Mix reagent (Applied Biosystems, Foster City, CA, USA). The forward and reverse primer sequences for Zn-SOD, Mn-SOD, CAT and GPx were obtained from published nucleotide sequences (Xiong* et al* 2010). The reactions were performed using the ABI Prism 7300 Sequence Detector (Applied Biosystems) under the following conditions: 50°C for 2 min, 95°C for 10 min, and 40 cycles of 95°C for 15 s and 60°C for 1 min. The specificity of the products obtained was confirmed by an analysis of the dissociation curves of the amplified product. As an internal control, the expression of the housekeeping gene 18S was used. The data obtained were analyzed using the comparative C_T_ method. All analyses were performed in triplicate.

#### 2.8.4. Statistical Analysis

The data was subjected to Kolmogorov-Smirnov normality tests and all data showed a normal distribution. All values are expressed as the mean ± standard error of the mean (SEM). Statistical analysis was performed using one-way analysis of variance (ANOVA), with Bonferroni posttest. Prism 5.0 (GraphPad, La Jolla, CA, USA) was used to perform the analysis. Differences were considered significant when p<0.05. For all analyzes the comparisons were C x C600; C x C1200; C x D; D x D600; D x D1200.

## 3. Results 

### 3.1. *In Vitro* Assays: Toxicity and DPPH Radical-Scavenging Activity

The concentrations of* B. trimera* extract and that of the positive control, Lapachol, which could kill 50% of the* A. salina *nauplii (LD_50_) (Table 1). By analyzing the data, it was possible to verify that the LD_50_ of the hydroethanolic extract of* Baccharis trimera* is equal to 924.60 *μ*g/ml, and the LD_50_ of the Lapachol substrate was 186.20 *μ*g/ml.

The ability of a given sample to reduce the absorbance of DPPH is indicative of the capacity of the sample to neutralize free radicals.  Table 2 shows the ability of* B. trimera* to neutralize the DPPH radical. The* B. trimera* extract displayed antioxidante activity at elevated concentrations (28.09%) and is less potent than the reference compound, BHA (87.37%).

### 3.2. *In Vivo* Assays

#### 3.2.1. *B. trimera* Improves Glycemic Profile

The glycemia of diabetic animals (D, D600 and D1200) was higher than the control animals (C, C600 and C1200), both at the beginning and at the end of treatment, proving that alloxan was effective in inducing type 1 diabetes (Table 3). The nontreated and treated diabetic groups (D, D600 and D1200) did not show significant differences if we observed between them; however, when we observed this parameter in relation to the initial and final time, we can note that* B. trimera* was able to decrease glycemia when the animals were treated with 600 mg/Kg (D600). In addition, there was a decrease in insulin in diabetic group, but* B. trimera* was able to increase this parameter in the concentration of 1200 mg/Kg (D1200). It can still be observed that the diabetic animals (D, D600 and D1200) presented weight loss at the end of 7 days of experiment (Table 3).

#### 3.2.2. Effect of* B. trimera* on Biochemical Parameters

There is a notable difference in the lipid profile, a significant increase in triacylglycerol levels was observed in the diabetic group (D) when compared to nondiabetic group (C). For this parameter, no difference was observed between the diabetic group treated with* B. trimera* and the nontreated diabetic group. Also no significant difference in total cholesterol and HDL fraction was observed in the groups assessed (Table 4).

Significant increase in urea levels in diabetic animal was noted but in contrast, and creatinine levels did not vary between any of the experimental groups. In relation to liver function, we observed a significant increase in ALT and AST activity in diabetic animals. Treatment with* B. trimera* was ineffective in improving kidney function, but was able to decrease AST activity in both tested concentrations (600 and 1200 mg/kg), improving liver function in diabetic animals (Table 4).

#### 3.2.3. Effect of* B. trimera* in Antioxidant Profile

**Figure 1:**
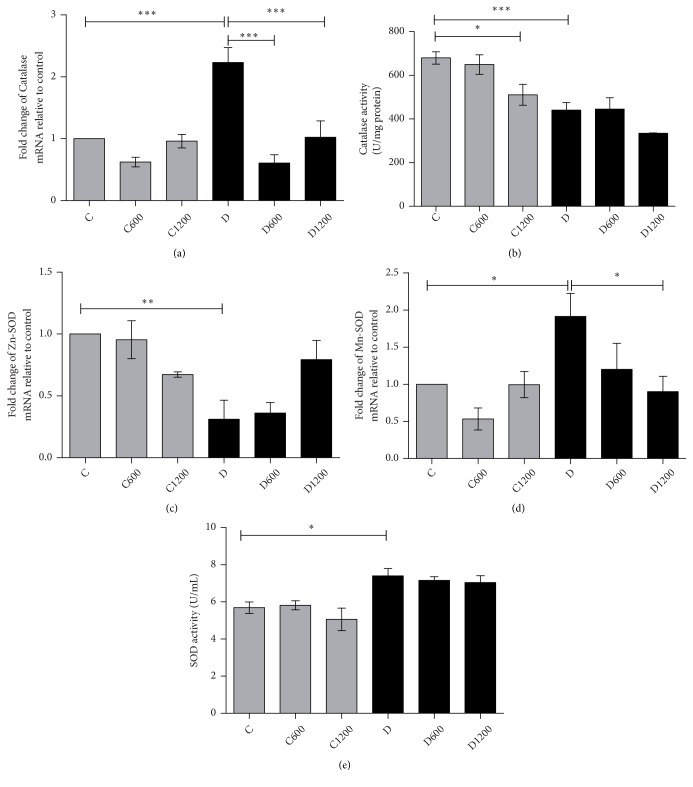
Effect of* Baccharis trimera *hydroethanolic extract on mRNA expression of liver catalase (a) and its activity (b); on mRNA expression of liver Zn-SOD (c), Mn-SOD (d), and its activity (e) in the livers of rats. C: control group; C600: treated control with 600 mg/Kg of extract; C1200: treated control with 1200 mg/Kg of extract; D: non treated diabetic group; D600: treated diabetic with 600 mg/Kg of extract; D1200: treated diabetic with 1200 mg/Kg of extract. Values are expressed as means ± SEM. The statistical analysis was performed between the groups: C x C600; C x C1200; C x D; D x D600; D x D1200. (*∗*) = p < 0.001; (*∗∗*) = p < 0.01; (*∗∗∗*) = p < 0.05.

The antioxidant status of the animals treated and nontreated was evaluated based on SOD, CAT, GPx and GR activity, total glutathione, reduced and oxidized glutathione, besides mRNA of these enzymes. Our results show that there was an increase in gene expression of catalase in the diabetic group (Figure 1(a)); however the enzyme was slightly less active (Figure 1(b)). Upon treatment with* B. trimera *extract in both concentrations, it promoted a decreased in gene expression of catalase in the diabetic animals when compared with nontreated diabetic group (Figure 1(a)), but no change in enzyme activity was observed in treated-diabetic groups (Figure 1(b)).

The nontreated diabetic animals displayed a decrease in the gene expression of Zn-SOD (Figure 1(c)) and an increase in Mn-SOD (Figure 1(d)). The activity of SOD was greater than in the nontreated diabetic group when compared control animals (Figure 1(e)). The* B. trimera *extract in 1200 *μ*g/ml decreased gene expression of Mn-SOD (Figure 1(d)).

**Figure 2:**
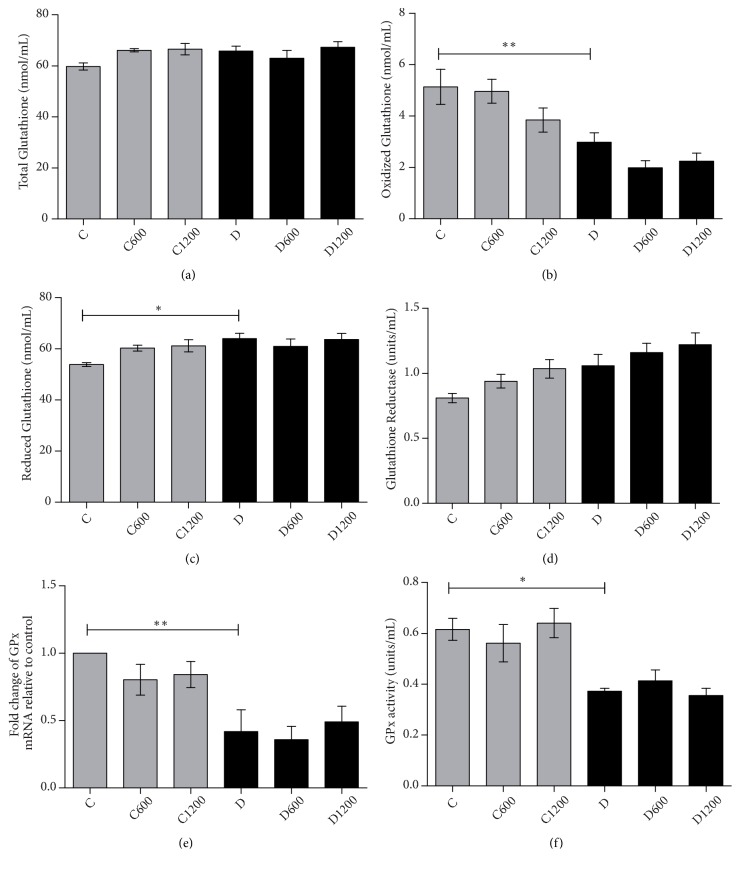
Effect of* Baccharis trimera *hydroethanolic extract on total glutathione (a), oxidized glutathione (b), reduced glutathione (c), activity of glutathione reductase (d), mRNA expression of glutathione peroxidase (GPx) (e), and activity of GPx (f) in the livers of rats. C: control group; C600: treated control with 600 mg/Kg of extract; C1200: treated control with 1200 mg/Kg of extract; D: non treated diabetic group; D600: treated diabetic with 600 mg/Kg of extract; D1200: treated diabetic with 1200 mg/Kg of extract. Values are expressed as means ± SEM. The statistical analysis was performed between the groups: C x C600; C x C1200; C x D; D x D600; D x D1200. (*∗*) = p < 0.001; (*∗∗*) = p < 0.01; (*∗∗∗*) = p < 0.05.

Diabetic animals showed no significant change in the total glutathione levels when compared to the control group (Figure 2(a)). Analysis of oxidized glutathione in nontreated diabetic animals showed lower levels of GSSG than their nondiabetic counterparts (Figure 2(b)). An increase in reduced glutathione in nontreated diabetic group was also found (Figure 2(c)). In order to assess the conversion of reduced glutathione to its oxidized form and the “recycling” of oxidized glutathione to its reduced state, the activity and expression of mRNA, glutathione peroxidase (GPx), and glutathione reductase were evaluated. The activity of GPx is lower in nontreated diabetic animals (Figure 2(f)) and this result was further confirmed by reduced expression of mRNA of this enzyme (Figure 2(e)). In contrast, glutathione reductase activity was unaltered in all the groups evaluated (Figure 2(d)). Treatment with the de* B trimera *extract did not alter glutathione.

#### 3.2.4. *B. trimera* Decrease Oxidative Damages

**Figure 3:**
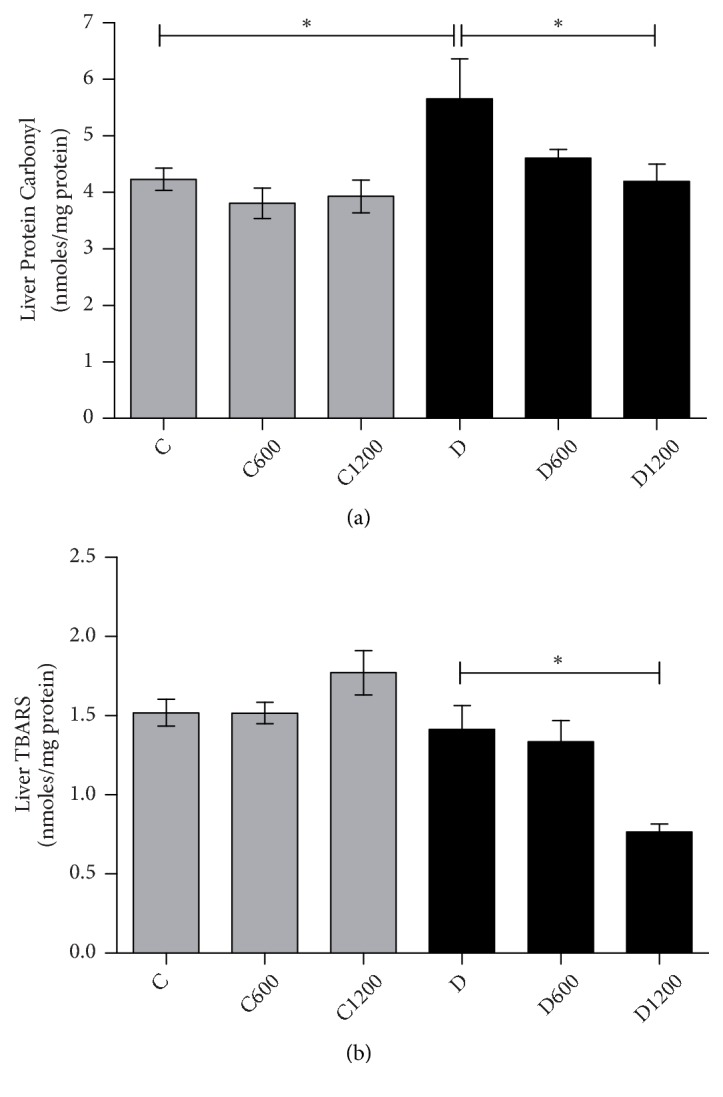
Effect of* Baccharis trimera *hydroethanolic extract on the level of carbonyl protein (a) and TBARS (b) in the livers of rats. C: control group; C600: treated control with 600 mg/Kg of extract; C1200: treated control with 1200 mg/Kg of extract; D: non treated diabetic group; D600: treated diabetic with 600 mg/Kg of extract; D1200: treated diabetic with 1200 mg/Kg of extract. Values are expressed as means ± SEM. The statistical analysis was performed between the groups: C x C600; C x C1200; C x D; D x D600; D x D1200. (*∗*) = p < 0.001; (*∗∗*) = p < 0.01; (*∗∗∗*) = p < 0.05.

To evaluate biomarker of oxidative damage TBARS and carbonyl protein levels were analysed. The diabetic group demonstrated an increase in carbonyl protein levels, when compared with control group (Figure 3(a)), but not altered TBARS (Figure 3(b)). A significant reduction in these parameters was observed when the animals received 1200 mg/kg of* B. trimera* extract.

## 4. Discussion

Plants have long been considered valuable sources of medicines for treating a variety of diseases and aliments [29]. Most, if not all, people have used plants as curatives. New estimates suggest that, in many developing countries, much of the population depends heavily on traditional practitioners and medicinal plants to meet their primary health care needs [30, 31]. Currently, medicinal products containing “carqueja” are marketed for different uses, including as an antidiabetic, both in countries where carqueja originates and in the United States and Europe [32]. However, the majority of medicinal plants have not been studied in terms of their toxic potential [33]. In this regard, the cytotoxic activities of the* B. trimera* hydroethanolic extracts were studied using the brine shrimp lethality bioassay, a test that permits the determination of the LD50, and the lethal dose capable of reducing the shrimp population by 50%. LD50 values above 1000 *μ*g/ml for natural compounds are considered nontoxic and values below 130 *μ*g/ml are classed as very toxic and lethal [25, 34, 35]. The hydroethanolic extract of* Baccharis trimera* provided a DL50 value of 924.60 *μ*g/ml and is therefore considered weakly toxic. In comparison, our positive control Lapachol is toxic and gave DL50 value of 186.20 *μ*g/ml.

It is known that* B. trimera* contains many bioactive compounds such as flavonoids and that these are well-known for their antioxidant activity [17, 36].* B. trimera* extract showed antioxidant activity, especially at high concentrations, but this activity was unremarkable when compared to the reference antioxidant butyl-hydroxyanisole (BHA). After the* in vitro* tests, the next step was to evaluate the antioxidant and antidiabetic effects of the* B. trimera* hydroethanolic extract in rats diabetic model. For this, was administered intraperitoneal alloxan, a diabetogenic drug that cause destruction of pancreatic *β*-cells by production of hydroxyl radicals and, thus, creates DM [37]. It is know that alloxan causes hyperglycemia, however, hypoglycemia may occur within 48 hours, presumably due to release of preformed insulin from damaged beta cells [37]. Thus, initial glycemia was measured 72 hours after the induction of diabetes so that it was not influenced by this effect. The results showed efficiency in the induction of DM, since glycemia remained above 300 mg/dl in all diabetic animals when compared with nondiabetic groups. The same profile was maintained in relation to final glycemia. In addition, diabetic animals showed a significant decrease in insulin levels compared with nondiabetic animals.* Baccharis trimera* was able to decrease glycemia and increase insulin after 7 days of treatment. A hypoglycemic also found when diabetic animals were treated with* B. trimera* aqueous extract for 7 days [12]. Probably, this effect may be associated with the presence of flavonoids and chlorogenic acids, as their hypoglycemic activity has been previously demonstrated [38]. Insulin is responsible for the signalization for internalization of glucose in cells to be used as an energy source. In the absence of this hormone, there are increased lipolysis and consequent weight loss [39]. This justifies the weight body loss found in diabetic animals after seven days treatment.

When the lipid profile, liver and renal function were evaluated, the results indicate a significant increase in the concentrations of triacylglycerol, urea AST, and ALT in the serum of diabetic animals compared to controls, demonstrating that 7 days of diabetes were sufficient to alter these parameters. These results demonstrate that the experimental diabetes model used here caused changes in plasmatic biomarkers that match the glucolipotoxicity of diabetes (hyperglycemia and higher levels of TAG) [30], in addition to altering renal and liver function. This is characteristic of an experimental model for type 1 diabetes because the development of kidney disease and dyslipidemia is common over the course of untreated diabetes [3].* B. trimera* did not promote change in lipid profile or renal function; however, the extract was able to decreased AST activity, showing improvement in liver profile. The hydroethanolic extract of* B. trimera* ameliorates a lipid profile and liver function in alcoholic fatty liver disease [13]. This may suggest that the mechanism of action of* B. trimera* depends on the model of disease studied.

Although intracellular glucose is metabolized primarily by glycolysis, excess intracellular glucose is subject to metabolism by alternative pathways under diabetic conditions. The accumulation of metabolites of these pathways plays an important role in diabetic complications, including the oxidative stress [40]. In this way, there is evidence that supplementation with a wide range of antioxidants may reduce oxidative stress in diabetics [41]. The protective action of several herbal medicines and their active constituents occurs through antioxidant enzymes (e.g., SOD, CAT, GPx, and GR), which maintain the pro-oxidant/antioxidant balance in the body. To eliminate ROS from the cellular system, SOD and CAT function cooperatively to remove superoxide radicals [42]. With respect to CAT expression, liver tissue from diabetic rats showed increased CAT expression compared to control animals. However, CAT enzyme activity was significantly decreased, suggesting that post-transcriptional modifications occur in diabetes. Regarding SOD, an increase in the mRNA expression of Mn-SOD and a decreased in Zn-SOD were observed. It is know that in diabetes mellitus main sources of oxidative stress are mitochondria [3]. Maybe this explains the increased in expression of Mn-SOD, a mitochondrial isoenzyme, which is probably leading to increased activity of SOD in diabetics animals. Although SOD is an antioxidant enzyme, some studies have suggested that it's over expression is in fact harmful to cells [43], mainly for pancreatic *β*-cells that are poor in enzymes that inactivate the H_2_0_2_ formed by SOD [44].

The GSH/GSSG ratio is a very important redox system in cells, since GSH aids the removal of ROS and protects thiol groups in macromolecules [45]. Normally, glutathione is found mainly in reduced form (GSH), while its oxidized form (GSSG) is much lower [46]. However, during oxidative stress these values are significantly altered, thus determining the GSH/GSSG ratio provides information on the redox status of the cells. In our study, we found no alteration in the concentration of total glutathione in the liver of diabetic rats when compared to control rat liver; however, a reduction in GSSG levels and an increase in GSH levels in diabetic rats were observed. Probably, this effect occurred because GPx activity was decreased in the liver of diabetic groups, while GR was not altered. It is known that GSH is cofactor of GPx [46], since a decrease in the activity of this enzyme was observed, consequently there was reduction of the use of the substrate, which was accumulated. In the same way, there was decrease of the GPx product (GSSG). The toxic effect of ROS that has been observed in many cells with over expressed SOD has been linked to elevated levels of H_2_O_2_ and the accompanying oxidative damage following hydroxyl radical formation [3]. The implication for SOD up-regulation is the high turnover of H_2_O_2_.

**Figure 4:**
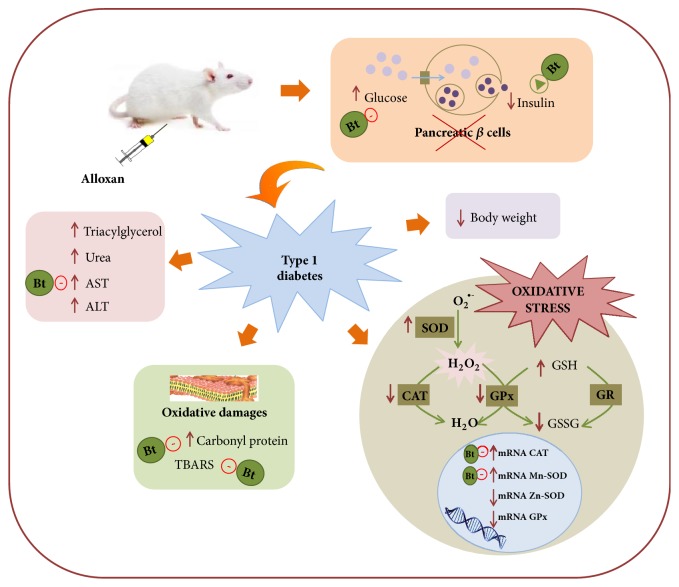
Type 1 diabetes induced by alloxan and the interference of* Baccharis trimera *hydroethanolic extract (Bt) in this process. When the diabetes model was induced with alloxan the animals had increased glucose, decreased insulin and body weight. In diabetic groups also found an increase in triacylglycerol, urea, AST and ALT. In the liver, there was an increase in SOD activity and decreased in CAT and GPx activity; there was an increase in GSH and decreased in GSSG. Besides, mRNA-CAT and mRNA-Mn-SOD were increased and mRNA-Zn-SOD and mRNA-GPx were decreased. Increased in carbonyl protein in the liver was also found. These events promote oxidative stress.* Baccharis trimera* (Bt) promoted a decrease in glycaemia, AST, carbonyl protein, and TBARS, mRNA-CAT, and mRNA-Mn-SOD and also an increase in insulin. AST: aspartate aminotransferase; ALT: alanine aminotransferase; SOD: superoxide dismutase; CAT: catalase; GPx: glutathione peroxidase; GR: glutathione reductase; GSH: reduced glutathione; GSSG: oxidized glutathione; TBARS: thiobarbituric acid reactive substances.

CAT and GPx are an endogenous enzymes that inactivate H_2_O_2_ and need to be replenished, to avoid the continuous formation of H_2_O_2_ overwhelming these enzyme. The enhanced activity of SOD and reduced CAT and GPx activity might generate excessive H_2_O_2_, which could give rise to other ROS such as hydroxyl radicals, thereby contributing to the oxidative stress in the liver of diabetic rats [3, 4, 47]. This hydroxyl radical is highly reactive and rapidly abstracts hydrogen atoms from biomolecules around it [47]. The inefficiency in the antioxidant response may justify the increase in the carbonylated protein levels observed in diabetes animals. In summary, we can conclude that diabetes causes alterations to the antioxidant capacity of the animals, through the regulation of the gene expression, activity of antioxidant enzymes, and oxidative damages in diabetic animals. Treatment with* B. trimera* altered CAT and Mn-SOD mRNA transcription, but no change in the activity of these enzymes was observed in either treatment with* B. trimera*, suggesting that the extract could act by regulating mRNA levels, specially trying to contain mitochondrial damage by the regulation of Mn-SOD, but not the activity of these enzymes. In addition,* B. trimera* ameliorates carbonylated protein and TBARS levels. Improvement in lipid peroxidation was also found when mice received hydroethanolic extract of* B. trimera* [13]. These results (summarized in Figure 4) suggest that although carqueja does not act on the antioxidant enzymes, it modulates their expression and decreases the oxidative damage promoted by diabetes. In conclusion, these results suggest that metabolic profile and redox status improves in diabetics animals treated with* B. trimera*.

## Figures and Tables

**Table 1:** Brine shrimp toxicity test for *Baccharis trimera* and controls (Lapachol and saline solution).

**REFERENCES Test**		**LC50 (** ***μ*** **g/mL)**
*Baccharis trimera*		924.6
Controls	Lapachol	186.2
	Saline solution	> 1.000

**Table 2:** Ability of *B. trimera* and the standard antioxidant butyl-hydroxyanisole (BHA) to neutralize the DPPH radical. Different concentrations (480, 240, 180, 120, 60, and 30 *μ*g/mL) of tested compounds were used.

	**Antioxidant Activity (**%**)**					
**Concentration (** ***μ*** **g/mL) **	**480**	**240**	**180**	**120**	**60**	**30**
BHA	87.37	84.58	82.44	65.87	30.96	11.91
*Baccharis trimera*	28.09	24.48	13.47	1.93	1.63	1.47

**Table 3:** Evaluation of initial and final glycaemia, initial and final weight, and insulin in nondiabetic and diabetic rats treated or not with hydroethanolic extract of *Baccharis trimera*. C: control group; C600: treated control with 600 mg/Kg of extract; C1200: treated control with 1200 mg/Kg of extract; D: non treated diabetic group; D600: treated diabetic with 600 mg/Kg of extract; D1200: treated diabetic with 1200 mg/Kg of extract. Values are expressed as means ± SEM. The statistical analysis was performed between the groups: C x C600; C x C1200; C x D; D x D600; D x D1200. Different letters indicate statistically significant between the compared groups (p< 0.05). Asterisk (*∗*) indicates differences between the same group, but in relation of initial and final parameter.

**Parameters**	**Experimental groups**					
	**C**	**C600**	**C1200**	**D**	**D600**	**D1200**
**Initial glycemia (mg/dL) **	94.88 ± 2.7^b^	84.38 ± 2.9^b^	93.0 ± 2.8^b^	471.9 ± 31.5^a^	497.6 ± 42.2^a*∗*^	400.7 ± 42.1^a^
**Final glycemia (mg/dL) **	94.97 ± 13.9^b^	100.4 ± 7.7^b^	99.33 ± 5.7^b^	435.3 ± 55.1^a^	386.8 ± 33.3^a*∗*^	403.9 ± 26.0^a^
**Initial body weight (g) **	177.9 ± 2.6	177.1 ± 2.3	178.2 ± 2.1	185.4 ± 1.7^*∗*^	184.8 ± 1.4^*∗*^	183.8 ± 2.3^*∗*^
**Final body weight (g) **	170.8 ± 4.0	169.7 ± 2.2	168.1 ± 2.5	160.6 ± 3.7^*∗*^	165.1 ± 6.0^*∗*^	161.2 ± 3.1^*∗*^
**Insulin (nmol/L) **	0.8579 ± 0.07^b^	0.8618 ± 0.02^b^	0.8621 ± 0.03^b^	0.6479 ± 0.04^c^	0.754 ± 0.05^c^	0.8792 ± 0.08^a^

**Table 4:** Determination of biochemical parameters after seven days of treatment with hydroethanolic extract of *Baccharis trimera*. C: control group; C600: treated control with 600 mg/Kg of extract; C1200: treated control with 1200 mg/Kg of extract; D: non treated diabetic group; D600: treated diabetic with 600 mg/Kg of extract; D1200: treated diabetic with 1200 mg/Kg of extract. Values are expressed as means ± SEM. The statistical analysis was performed between the groups: C x C600; C x C1200; C x D; D x D600; D x D1200. Different letters indicate statistically significant between the compared groups (p < 0.05).

**Parameters**	**Experimental groups**					
	**C**	**C600**	**C1200**	**D**	**D600**	**D1200**
**Triacylglycerol (mmol/L)**	0.6 ± 0.05^b^	0.5 ± 0.03^b^	0.5 ± 0.02^b^	1.04 ± 0.2^a^	1.04 ± 0.1^a^	1.1 ± 0.1^a^
**Total cholesterol (mmol/L) **	1.6 ± 0.08	2.08 ± 0.2	1.9 ± 0.1	1.08 ± 0.2	1.9 ± 0.2	1.6 ± 0.07
**HDL (mmol/L) **	1.2 ± 0.1	1.1 ± 0.1	1.06 ± 0.09	0.9 ± 0.08	0.9 ± 0.1	0.8 ± 0.1
**Urea (mmol/L) **	9.6 ± 0.2^b^	9.7 ± 0.3^b^	8.3 ± 0.4^b^	29.6 ± 1.6^a^	28.7 ± 1.6^a^	25.8 ± 2.06^a^
**Creatinine (mmol/L) **	60.3 ± 2.9	62.3 ± 1.8	63.4 ± 3.9	72.5 ± 2.2	88.7 ± 4.01	73.9 ± 6.08
**AST (U/mL)**	107.1 ± 3.9^b^	116.2 ± 4.9^b^	113.2 ± 3.6^b^	139.1 ± 4.6^a^	113.1 ± 5.4^c^	112.7 ± 6.9^d^
**ALT (U/mL) **	46.7 ± 1.8^b^	48.6 ± 2.3^b^	50.2 ± 2.8^b^	93.9 ± 6.3^a^	86.2 ± 8.5^a^	91.9 ± 8.3^a^

## Data Availability

The data used to support the findings of this study are available from the corresponding author upon request.
